# The role of birthplace and educational attainment on induced abortion inequalities

**DOI:** 10.1186/s12889-016-3984-y

**Published:** 2017-01-13

**Authors:** Yolanda González-Rábago, Elena Rodriguez-Alvarez, Luisa N. Borrell, Unai Martín

**Affiliations:** 1 Department of Sociology 2, University of the Basque Country UPV/EHU, Barrio Sarriena s/n 48940, Leioa, Spain; 2 Department of Nursing I, University of the Basque Country UPV/EHU, Barrio Sarriena s/n 48940, Leioa, Spain; 3 Department of Epidemiology & Biostatistics, Graduate School of Public Health & Health Policy, City University of New York, New York, NY USA; 4 Social Determinants of Health and Demographic Change - Opik, Leioa, Spain

**Keywords:** Induced abortion, Immigration, Education, Inequalities, Repeat induced abortion, Second trimester induced abortion

## Abstract

**Background:**

Induced abortion (IA) has shown social inequality related to birthplace and education with higher rates of IAs in immigrant and in less educated women relative to their native and highly educated counterparts. This study examined the independent and joint effects of birthplace and education on IA, repeated and IA performed during the 2nd trimester of pregnancy among women residing in the Basque Country, Spain.

**Methods:**

We conducted a cross-sectional population-based study of IA among women aged 25–49 years residing in the Basque Country, Spain, between 2011 and 2013. Log-binomial regression was used to quantify the independent and joint effects of birthplace and education attainment on all outcomes.

**Results:**

Immigrant women exhibited higher probability of having an IAs (PR: 5.31), a repeated (PR: 7.23) or a 2nd trimester IAs (PR: 4.07) than women born in Spain. We observed higher probabilities for all outcomes among women with a primary or less education relative to those with a graduate education (All IAs PR: 2.51; repeated PR: 6.00; 2nd trimester PR: 3.08). However, no significant heterogeneity was observed for the effect of education on the association of birthplace with IAs, repeated or 2nd trimester IAs.

**Conclusions:**

Birthplace and education are key factors to explain not only an IA decision but also having a repeated or a 2nd trimester IA. However, the effects of birthplace and education may be independent from each other on these outcomes. A better understanding of these factors on IAs is needed when designing programs for sexual and reproductive health aimed to reduce inequalities among women.

## Background

There is consensus on the existence of inequalities in health generated by unequal distribution of social determinants of health [1, 2]. For example, factors considered as social determinants of health such as gender, birthplace or social position indicators determine access to opportunities and resources related to health [3]. Specifically, several studies show health inequalities by birthplace as a result of unequal distribution of factors considered as social determinants of health such as social class [4, 5]. In addition, previous studies show that education, an indicator of social class, is an important social determinant of health inequalities with a clear gradient [6–8]. Hence, birthplace and education are indicators of population health inequality, especially reproductive health [9]. Furthermore, these indicators could not only act independently but also together to affect the health of the individual [1, 10]. The intersection of the different social determinants of health has been examined especially in social sciences as related to gender and immigration [11–13]. Recently, this approach has been used in health studies, specifically on inequalities of self-perceived health [10, 14–16]. However, fewer studies have examined the combined effects of two or more social determinants on reproductive health [17, 18].

Induced abortion (IA), an indicator of reproductive health, has been associated with both to birthplace [19–24] and educational attainment [25–28]. Studies examining IA and birthplace suggest that compared to natives women, immigrant women exhibited higher IA rates, number of repeated and IAs performed during the second trimester of pregnancy [21–24]. These outcomes are also more common among less educated women than among highly educated women [25–28]. Both repeated and IAs performed during the 2nd trimester pose greater risks for women’s health and have been linked to adverse outcomes in future pregnancies [25, 29–31]. Thus, it is important not only to examine the prevalence of IAs but also the inequalities of IAs associated with birthplace and education, independently and jointly, to design and implement policies to reduce such inequalities.

During the last two decades Spain has experienced a tremendous growth of its immigrant population, placing Spain among the European countries with the highest percentage of foreign-born population (12.6%) in 2015 [32]. In Spain, the public health system coverage is considered universal de facto, with the law regulating access to IA and related services. In the Basque Country, access to IA is guaranteed through both public and private centers [33]. In 2012, Spain introduced the Royal Decree Law 16/2012 limiting access to health services to illegal immigrants. To mitigate this Decree, the Basque Country introduced the Decree 114/2012 to ensure health care access and coverage to this population. Thus, unequal access to the health system, specifically related to reproductive health, between immigrant and native population, makes imperative to examine inequalities on IA outcomes. To address this gap, this study aims to examine the independent and joint effects of birthplace and education on IA, repeated and IAs performed during the 2nd trimester among women residing in the Basque Country, Spain, during 2011 and 2013.

## Methods

This study was a cross-sectional population-based of IAs among women aged 25 to 49 years residing in the Basque Country, Spain, between 2011 and 2013. Information on IAs came from the Department of Health of the Basque Government, Registry of Induced Abortions, and included all IAs from women residing in the Basque Country. The data were collected through notification forms by accredited centers to conduct IAs submitted periodically records to the Registry of Abortions. Validation, encryption and process were performed by the Registry according to established protocols by the Ministry of Health, Social Services and Equality. In addition, we used data for women residing in the Basque Country collected by the Population and Housing Census 2011 according to age, place of birth and educational attainment.

The dependent variables were all IAs, repeated IAs and IAs conducted during the 2nd trimester among women who have an IA. A repeat IA refers to women who have a previous IA, while a 2nd trimester IA was an IA performed at ≥12 weeks of gestational age of pregnancy [29]. The independent variables were educational attainment, specified as primary or less, secondary, and graduate; and birthplace, classified women as born in Spain or born in countries with a Human Development Index (HDI) <0.78 in 2011 [34] (hereafter, the latter would be referred as low income countries). Thus, birthplace refers to women born in low income countries because women from high HDI countries have a similar or better socioeconomic position than native women, making it difficult to rule out inequalities between natives and immigrant women [15]. Variables considered as covariates were year of the intervention and age groups (25–29, 30–34 and 35–49). We focus the analyses on women 25 years and older to avoid including women who are still in school as the latter may lead to residual confounder.

Out of the 11,946 women with an IA during 2011 and 2013, we excluded those IAs conducted for fetal risk and rape (*n* = 596); those IAs among women born in high income countries (*n* = 216); records of women younger than 25 years of age (*n* = 3,388), without information on educational attainment (*n* = 516), and number of IA (*n* = 1); or for those reporting employment status as a pensioner (*n* = 18). These exclusions resulted in 7,211 records for analysis purposes.

### Statistical analysis

Prevalence estimates and associations of all IAs as well as repeated and 2nd trimester IAs with birthplace and educational attainment were calculated using as the denominator data obtained from the Population and Housing Census 2011 before and after adjusting for age and year of IA intervention. Log binomial regression was used to calculate the prevalence ratios (PR) and corresponding 95% confidence intervals (CI) for the association of birthplace and educational attainment with each dependent variable before and after controlling for age and year of the IAs. For the final model, the association for birthplace was further adjusted for education while the one for education was adjusted for birthplace. To determine the joint effect of birthplace and educational attainment, interaction terms were examined in the final models for each outcome.

Data management and analysis were conducted using SAS version 9.4 for Windows (SAS Institute Inc., Cary, NC).

## Results

For women with IAs in the Basque Country between years 2011 and 2013, roughly one third of IAs were performed during each year included in the study, over two thirds of IAs were performed among women younger than 34 years of age and over half among women with a secondary level of education (Table 1). Among women with IAs, over a third (36.1%) was repeat IAs whereas 4.2% were performed in the 2nd trimester. While these distributions remain the same regardless of birthplace, there were some differences (*p*-values <0.05): When compared to Spanish women, women born in low income countries were more likely to be younger than 34 years of age (60.9% vs. 71.2%), have a primary or lower education (7.9% vs. 30.5%), more likely to have repeat IAs (28.2% vs. 45.7%) and less likely to have IAs in the 2nd trimester (4.7% vs. 3.7%).

**Table 1:** Distribution of characteristics of women aged 25 to 49 years who have had an IA for the total population, repeat and second trimester of IAs: Basque Country, years 2011-2013

Characteristics	Total^a^ (*n* = 7211)	Spain	Low Income Countries	*P*-value**
		55.1 (3971)	44.9 (3240)	
Year of intervention				
2011	34.6 (2496)	35.3 (1401)	33.8 (1095)	0.21
2012	31.9 (2302)	32.1 (1275)	31.7 (1027)	
2013	33.5 (2413)	32.6 (1295)	34.5 (1118)	
Age (years)				
25–29	34.2 (2465)	31.7 (1260)	37.2 (1205)	<0.0001
30–34	31.3 (2261)	29.2 (1160)	34.0 (1101)	
35–39	23.8 (1715)	25.9 (1028)	21.2 (687)	
40–49	10.7 (770)	13.2 (523)	7.6 (247)	
Educational attainment				
Primary or less	18.1 (1303)	7.9 (315)	30.5 (988)	<0.0001
Secondary	56.3 (4061)	54.0 (2144)	59.2 (1917)	
Graduate	25.6 (1847)	38.1 (1512)	10.3 (335)	
Repeat IAs				
Yes	36.1 (2603)	28.2 (1122)	45.7 (1481)	<0.0001
No	63.9 (4608)	71.7 (2849)	54.3 (1759)	
Second trimester IAs				
Yes	4.2 (306)	4.7 (187)	3.7 (119)	<0.03
No	95.8 (6305)	95.3 (3784)	96.3 (3121)	

^a^Percentage (*n*)

**P-values for chi-square statistics

The prevalence of IAs was 6.0%, with 2.2% being repeat IAs and 0.3% being IAs performed in the 2nd trimester (Table 2). Regardless of the outcomes, women born in low income countries exhibited higher prevalence than among Spanish women. For educational attainment, the prevalence estimates of all outcomes were higher among women with a primary or lower education relative to those with a secondary or graduate level of education.

**Table 2:** Prevalence and prevalence ratios (PR) with their 95% confidence intervals (CI) associated with birthplace and educational attainment on induced abortions (IA), repeat and second trimester IAs: Basque Country, years 2011–2013

	Prevalence* (per 1,000)	Unadjusted PR (95% CI)	PR (95% CI)^a^	PR (95% CI)^b^	PR (95% CI)^c^
	*All IA*				
Birthplace					
Spain	3.6	1.00	1.00	1.00	1.00
Low income countries	29.1	7.94 (3.87, 16.28)	6.51 (3.15, 13.43)	6.51 (3.15, 13.45)	5.31 (2.28, 12.24)
Educational attainment					
Primary or less	17.7	4.47 (1.70, 11.79)	5.42 (2.22, 13.24)	5.42 (2.21, 13.28)	2.51 (0.92, 6.82)
Secondary	6.2	1.59 (0.57, 4.40)	1.89 (0.73, 4.86)	1.89 (0.73, 4.87)	1.41 (0.56, 3.57)
Graduate	3.9	1.00	1.00	1.00	1.00
Total	6.0				
	*Repeat IA*				
Birthplace					
Spain	1.2	1.00	1.00	1.00	1.00
Low income countries	15.4	13.04 (6.28, 27.08)	10.71 (5.13, 22.38)	10.71 (5.12, 22.42)	7.23 (2.89, 18.09)
Educational attainment					
Primary or less	9.0	12.65 (4.78, 33.48)	15.66 (6.29, 38.99)	15.66 (6.26, 39.16)	6.00 (2.10, 17.11)
Secondary	2.5	3.54 (1.28, 982)	4.25 (1.62, 11.13)	4.25 (1.62, 11.16)	2.92 (1.17, 7.29)
Graduate	0.7	1.00	1.00	1.00	1.00
Total	2.2				
	*Second Trimester IA*				
Birthplace					
Spain	0.2	1.00	1.00	1.00	1.00
Low income countries	1.1	6.37 (3.07, 13.20)	5.25 (2.51, 11.00)	5.25 (2.48, 11.13)	4.07 (1.70, 9.75)
Educational attainment					
Primary or less	0.8	4.73 (1.77, 12.65)	5.82 (2.32, 14.59)	5.82 (2.30, 14.74)	3.08 (1.08, 8.77)
Secondary	0.2	1.39 (0.50, 3.91)	1.65 (0.63, 4.33)	1.65 (0.62, 4.39)	1.32 (0.48, 3.61)
Graduate	0.2	1.00	1.00	1.00	1.00
Total	0.3				

* *P*-value for chi-square statistics for all comparisons of prevalence estimates ≥0.20

^a^Adjusted for age

^b^Adjusted for age and year of intervention

^c^Adjusted for age, year of intervention and education for country of birth and country of birth for education

Table 2 shows that for all IAs, women born in low income countries were more likely to have an IA than women born in Spain before and after adjustment. This association remained significant regardless of the variables controlled for. However, the greatest attenuation was observed in the fully adjusted model where women born in low income countries had a 5.31 (95% CI: 2.28, 12.24) greater probability of having an IA than women born in Spain. For education, women with a primary or lower education had a greater probability of having an IA than women with a graduate level of education before and after controlling for a) age and b) age and year of intervention. However, this association was no longer significant when further adjusting for birthplace. Similarly, the associations of birthplace and education with repeated and 2nd trimester IAs exhibited a similar pattern than the ones observed for all IAs and remained significant regardless of the variables controlled for. However, this association was significant and stronger for repeated IAs (crude PR for birthplace: 13.04 and adjusted PR for age, year of intervention and education: 7.23). For 2nd trimester IAs, these estimates were 6.37 (95%CI: 3.07, 13.20) and 4.07 (95%CI: 1.70, 9.75) for the unadjusted and fully-adjusted, respectively. For education, women with primary or lower education were 6.0 (95%:2.10, 17.11) and 3.08 (95%CI: 1.08, 8.77) times more likely to have a repeat and 2nd trimester IAs, respectively, than women with a graduate level of education after controlling for age, year of birth and birthplace. This association was also observed for repeated IAs for secondary education relative to graduate education (PR: 2.92; 95%CI: 1.17, 729). However, no association was observed between educational attainment and all IAs.

No heterogeneity of the association of birthplace with each outcome was observed according to education (*p*-interactions >0.50). However and despite the lack of a significant interaction, Table 3 shows that the probability for repeated and 2nd trimester IAs among women from low income countries appears to increase with education, with the highest probability observed among women with a graduate level of education.

**Table 3:** Prevalence and prevalence ratios (PR)^a^ with their 95% confidence intervals (CI) associated with country of birth on induced abortions (IA), repeat and second trimester IAs according to educational attainment: Basque Country, years 2011–2013

	Educational attainment		
	Primary or less	Secondary	Graduate
	*All IA*		
Birthplace			
Spain	1.00	1.00	1.00
Low income countries	4.89 (1.51, 15.84)	5.54 (1.62, 18.90)	4.54 (1.38, 14.94)
	*Repeat IA*		
Birthplace			
Spain	1.00	1.00	1.00
Low income countries	5.00 (1.47, 16.87)	7.38 (2.21, 27.04)	8.47 (2.57, 27.84)
	*Second Trimester IA*		
Birthplace			
Spain	1.00	1.00	1.00
Low income countries	3.33 (0.90, 12.34)	3.92 (1.04, 14.81)	4.97 (1.37, 18.04)

^a^Adjusted for age and year of intervention

Interaction between country of birth and education for delay IVE, *p*-value = 0.94; for repeat IVE, *p*-value = 0.84; for overall IVE, *p*-value = 0.96

Three-way Interaction for age group, country of birth and education for overall IVE, *p*-value = 0.99

## Discussion

Consistent with previous studies, we found an association between birthplace and IAs [19, 35–37] underscoring the importance of educational attainment on having IAs [20, 22, 28, 38]. Similarly, previous studies have examined the effect of birthplace and education on repeat IAs [21, 24, 27, 39]. For instance, Fisher et al. found that immigrant women were 83% more likely to have a repeat IA than Canadian women [21]. Finally, our findings concur with a study in Spain that found immigrant women being more likely to repeat IAs than native women [39].

In our study, repeated IA was associated with lower education with a probability of having a repeat IA in women with primary education than in those with a graduate education. Consistent with our findings, Makenzius et al., showed that women with a high school or lower education were 50% more likely to repeat an IA than women with higher education in Sweden [27]. Finally, while few studies have examined IAs conducted during the second trimester [23, 29], the evidence seems to corroborate our results. For example, in a Dutch study, immigrant women exhibited higher prevalence of IA in the second trimester than native women [23]. Moreover, Font-Ribera et al., found that low education seems to be a risk factor for IA in the second trimester in Spain [29].

As with previous studies [18, 20], we observed that repeated or 2nd trimester IA appears to increase with education among immigrant women. This finding may suggest that education may not be protective for IAs in immigrant women as it does for native women. Limited and evidence exists on this issue with two studies in Norway [18, 20] suggesting that in contrast to Norwegian women, a high level of education did not have a protective effect for immigrant women.

Our findings of increase of repeated and 2nd trimester IAs associated with education among immigrant women relative to native women lead us to seek potential explanations for this differential educational effect. First, a lower level of sexual education and a relationship less equal with partners (limiting a woman negotiating power on the sexual relationship) among immigrant women may explain the higher frequency of unintended pregnancies than among native ones, regardless of level of education. This argument has been suggested in the Norwegian context, noting a higher importance of cultural factors over education when it comes to reproductive decisions [18]. Second, once there is an unintended pregnancy among immigrant women, the decision to interrupt may be influenced by factors such as reduced social and family support in the host country as well as poorer employment opportunities and socioeconomic status. Thus, less social support [40] and lower occupational skills faced by immigrant women [41] could be more important than education on the decision of having an IA or not. The latter suggests that education as a social position indicator may be less important or that it could be intertwined with other social factors for immigrant women.

A limitation of this study is the use of data from a register. These data are limited, preventing the inclusion of variables relevant to understand the relationship of birthplace and educational attainment with IA, such as, the length of residence in Spain. Despite this limitation, registry data are less biased than data obtained from population-based interviews subject to recall bias or non-response rates (under- or over-reporting) [42, 43].

## Conclusions

Our findings contribute to the study of IA outcomes in our context, where few studies have examined such outcomes [26, 37]. In addition, this study goes a step further by examining the combined effects of immigration status and education attainment on IAs, repeated and 2nd trimester IAs, the effects are rarely examined nowadays despite the associated risks to women’s health. Moreover, this study called attention to the importance of understanding the factors explaining not only an IA decision but also having a repeated or 2nd trimester IA. A better understanding of these factors, independently and/or together, is needed when designing programs for sexual and reproductive health education and access to care that may reduce inequalities between immigrant and native populations. Furthermore, the development of research and practice strategies capturing inequalities of different social groups are imperative [44].
